# Organizing a Mass Gathering Amidst a Rising COVID-19 Public Health Crisis: Lessons Learned From a Chinese Public Health Forum in Vancouver, BC

**DOI:** 10.7759/cureus.12365

**Published:** 2020-12-29

**Authors:** Jayneel Limbachia, Hollis Owens, Maryam Matean, Sophia S Khan, Helen Novak-Lauscher, Kendall Ho

**Affiliations:** 1 Digital Emergency Medicine, University of British Columbia, Vancouver, CAN; 2 Department of Emergency Medicine, University of British Columbia, Vancouver, CAN

**Keywords:** mass gatherings, public health crisis, covid-19, pandemic, risk-mitigation, health promotion, chronic disease management, lessons learned, virtual conference, culturally specific

## Abstract

Introduction

The coronavirus disease 2019 (COVID-19) evolved from a rising public health concern to a pandemic over mere weeks. Before March 11, 2020, the Public Health Agency of Canada had not advised against any mass gatherings. Herein, we highlight practical precautions taken by event organizers to adapt to the rising public health threat from COVID-19 and maintain public safety when conducting a health forum for the Chinese community of Vancouver, British Columbia on February 22, 2020.

Materials and Methods

In the pre-forum phase, we advertised the availability of virtual conferencing for remote participation in the forum and also had an official communication from the Ministry of Health available regarding COVID-19 on our website. At the forum, we ensured that attendees sanitized their hands at registration and had access to sanitizers throughout the forum. Additionally, we provided translated health literature on COVID-19 to participants and had our health professional speakers address COVID-19-related questions.

Results

This year, 231 older Chinese adults attended the forum in-person, while 150 participated remotely. The total number of 381 participants compares well to previous iterations of the forum, with twice the amount of participants on average attending online than before. Of the participants who attended the forum, 89% suggested that the forum would be effective in improving their overall health and 87% cited the forum’s utility in directing them to access community resources. None of the attendees had COVID-19 or are suspected to have contracted it at the forum.

Conclusion

Conducting a mass gathering during a crisis required closely following guidance from local public health authorities, constant and clear communication with attendees, and employing practical risk mitigation strategies.

## Introduction

The novel coronavirus disease 2019 (COVID-19) has transformed the way we interact with others and has posed several challenges to our healthcare systems, including healthcare delivery. The World Health Organization (WHO) has identified several strategies and public health practices to curb the spread of COVID-19 and its impact on the global population, including maintaining a 1-metre distance and wearing masks in public [1]. In addition, the WHO has emphasized the need to avoid crowded spaces where close contact with others is inevitable, such as during a mass gathering [2]. A mass gathering is defined by the WHO as any public event which includes a conglomeration of people who have the potential to spread the infection and place a burden on the local health authorities’ ability to respond to that threat. Historically, mass gatherings have coincided with other disease-causing pathogens, such as the 2009 pandemic (pdm) influenza A (H1N1) or the Middle East respiratory syndrome-related coronavirus (MERS-CoV) and the 2013 Middle East respiratory syndrome (MERS) outbreak [3]. However, the exact role of such mass gatherings in disease transmission and spread has not been fully documented [3-4]. In addition, the implementation of appropriate public health measures at mass gatherings, based on the World Health Assembly’s message on preventive approaches to curbing infectious disease spread globally, has reduced the scale of the issue over the last few years [5].

In the context of COVID-19, the risk from mass gatherings remains high [6-7]. Several mass gatherings across the world have been linked to an increase in COVID-19 cases globally or locally, including religious and professional gatherings like the Vancouver Dental Conference [8-9]. However, few mass gatherings have been documented to have coincided with significant COVID-19 outbreaks. Even the one prominent hypothesized example of a mass gathering leading to a massive outbreak (i.e., the Carnival in Brazil) was not conclusive in determining whether the first case in the country had contact with people attending the mass gathering or if there were other carriers present in the country at the time [5, 10]. A more recent example of a mass gathering, in the form of the Black Lives Matter protests across the United States (US), has also not been conclusively identified as a super-spreader event, with other factors, such as infrastructure and population dynamics, contributing to a small increase in case counts [11]. While part of the reason for such a low number of mass gatherings leading to massive COVID-19 outbreaks may be attributable to a widespread preemptive cancellation of large-scale public events that ensued as soon as the pandemic was officially declared, there may be other factors that warrant further investigation [12]. Interestingly, in the case of COVID-19, the rapidly evolving situation and uncertainty have led governments and health authorities to largely focus on avoidance rather than conducting an independent assessment of the risks involved when it comes to mass gatherings [5]. While it is important to promptly adopt the least risk-averse options by preemptively canceling mass gatherings before research-informed interventions are considered [13], a careful risk evaluation and employing appropriate public health practices may reduce the risk of transmission, as seen with the success of the Hajj 2020 pilgrimage [8, 14]. Thus, despite the lack of clarity on the role of mass gatherings in the spread of COVID-19, it is reasonable to consider novel risk mitigation strategies that can be effectively employed in the wake of a public health crisis to reduce the impact of canceled mass gatherings on its beneficiaries.

The interCultural Online Health Network (iCON) program, funded by the BC Ministry of Health’s (BC MoH) Patients as Partners initiative, is a community engagement initiative that brings together healthcare practitioners, community members, patient advocacy groups, and media partners to disseminate health education surrounding chronic diseases among multicultural populations [15]. The program, which primarily caters to Chinese and Punjabi-speaking communities across BC, has hosted over 50 public health workshops and forums since its inception in 2007. iCON’s annual flagship health forum(s) for the Chinese and South Asian communities are notably popular events. Since the last two iterations of the forums, iCON has been bringing together a group of healthcare professionals, including physicians, nurses, dietitians, pharmacists, occupational therapists, and physiotherapists, who deliver culturally specific health education to patients and caregivers in their native language through panel discussions, presentations, and health screenings. These forums have attracted 300 to 400 participants on average and focus on aspects of chronic disease prevention and management at home under the theme of “Healthy @ Home.”

This year, the public health landscape preceding iCON’s 2020 Chinese Health Forum, which was scheduled to take place on February 22, 2020, was changing by the day. COVID-19 grew from a rising public health concern into a pandemic over mere weeks. The WHO declared COVID-19 as an official pandemic on March 11, 2020. However, before March 11, 2020, neither the WHO nor the Public Health Agency of Canada (PHAC) had published any guidelines that advised against mass gatherings [16]. In fact, the British Columbia (BC) Provincial Health Officer only restricted mass gatherings of more than 250 people on March 13, 2020, and subsequently, of more than 50 people later on March 16, 2020, as an evolving response to the growing concern over COVID-19. Contemporaneously, public events, such as the International Hongkong and Shanghai Banking Corporation (HSBC) Rugby Sevens tournament, which attracted thousands of attendees, did take place on March 7-8 at BC place, while the Vancouver Canucks games were canceled from March 12, 2020 onward. Most movie theatres also remained open and fully functional throughout Canada up until March 16. Prior to our event on February 22, 2020, there were only six cases of COVID-19 in BC, with only one of them not associated with recent travel to China. The risk to the public was judged to be low in BC at that time. The advice from BC’s provincial health authorities and the British Columbia Centre for Disease Control (BCCDC) around that time were to self-monitor symptoms, such as fever, cough, and difficulty breathing if the person had traveled to China [17]. People were simply being asked to wash their hands frequently, put on a mask, and report to a healthcare provider if they were symptomatic.

Over the years, iCON forums have highlighted the need for culturally specific health education and assistance in navigating the healthcare system among the multicultural populations of BC [15]. In addition to the knowledge delivered, forum participants have repeatedly appreciated the dissemination of take-home resources at the forum, which highlights both culturally relevant and language-appropriate tips on how to self-manage chronic diseases at home. Furthermore, the forums serve to bridge the gap that many multicultural patients and their caregivers face when accessing healthcare services by bringing all the allied healthcare providers and their beneficiaries under one roof [18]. Prior to the iCON 2020 Chinese Health Forum, several participants who had pre-registered for the forum contacted us and emphasized their reliance on such culturally specific health education, all the while citing their concern for safety from COVID-19. As healthcare practitioners and providers interested in improving the health of vulnerable populations, we acknowledged the need for this forum but also understood the gravity of organizing a mass gathering during such an unprecedented public health crisis. At the time, a completely virtual forum delivery option would have been difficult for our target audience of older Chinese adults, given their limited access to technology. Thus, employing a combination of in-person and online options with appropriate risk assessment based on local guidance in organizing our forum was well warranted. In this paper, we highlight the precautionary steps and risk mitigation strategies employed by our team in enabling the smooth implementation of the iCON Chinese Health Forum on February 22, 2020 in Vancouver, BC and upholding public interest in receiving culturally specific health education to improve their health.

## Materials and methods

Setting

In February 2020, the PHAC had not yet developed an official risk assessment tool with information on when to conduct or cancel mass gatherings to reduce the spread of COVID-19 (issued on March 11, 2020) [19]. At the time, official guidelines on screening or testing were also not available from any local health authorities or the BCCDC. Being closely associated with the BC MoH and partnered with the Vancouver Coastal Health Authority (VCHA), we were well-positioned to carry out the event based on our local risk assessment and guidance from the VCHA. The February 22, 2020 iCON Chinese Health Forum was scheduled to take place at the Chinese Cultural Center in downtown Vancouver without any cost to the public. Given the information at the time, we identified the risk involved based on the homogeneity of the local population attending, the little chance of intermingling throughout the event, and an indoor setting that could accommodate approximately 500 people. Our team assessed the need for an approach that would keep the attendees safe and informed but also encourage those who had traveled to China around the time to not attend the forum in-person. 

Strategy

**Figure 1:**
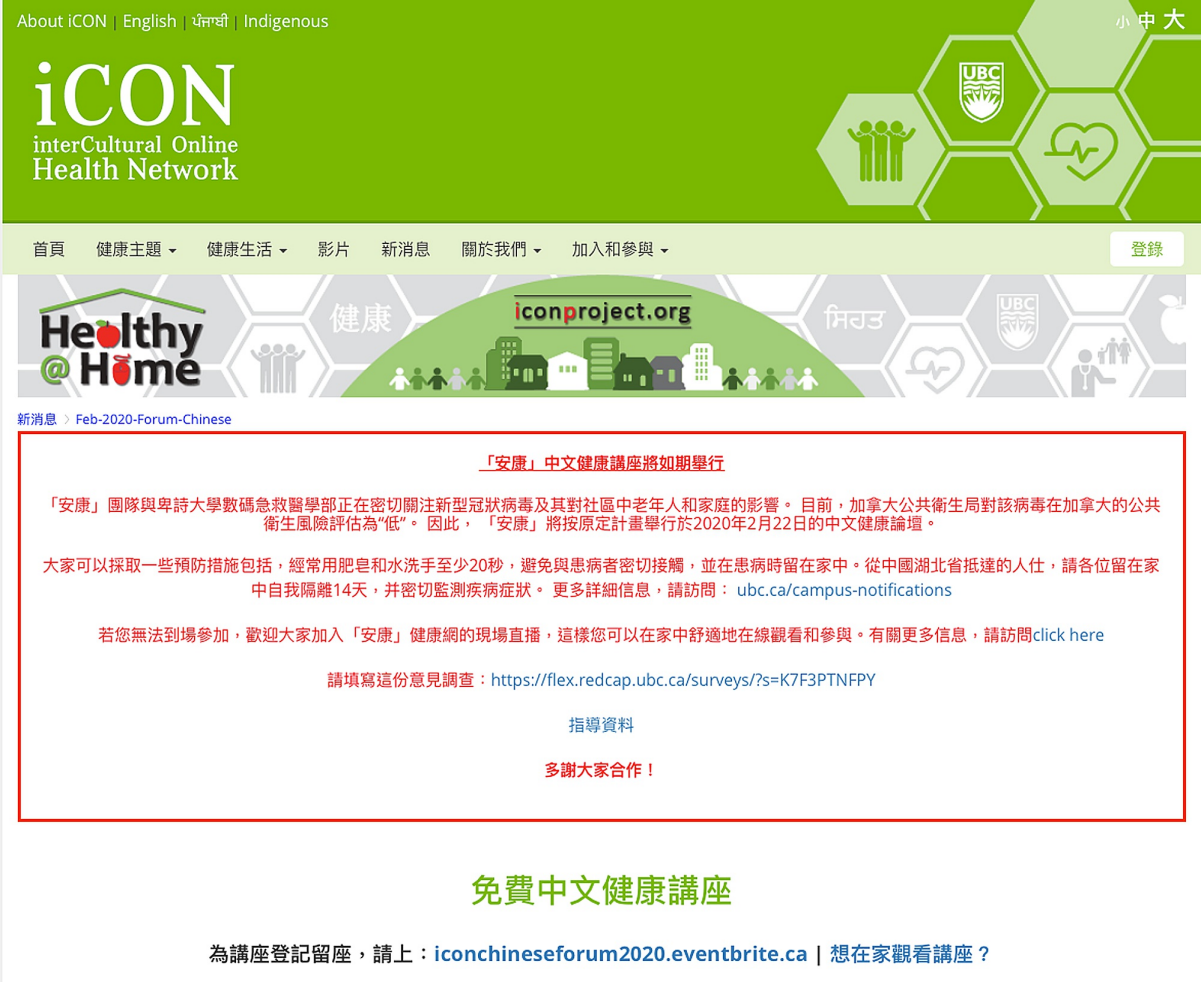
The traditional Chinese version of the official communication on COVID-19 developed in partnership with the British Columbia Ministry of Health for prospective forum attendees hosted on the interCultural Online Health Network's website Derived from the interCultural Online Health Network's website in February 2020. Available for public use. COVID-19: coronavirus disease 2019

**Figure 2:**
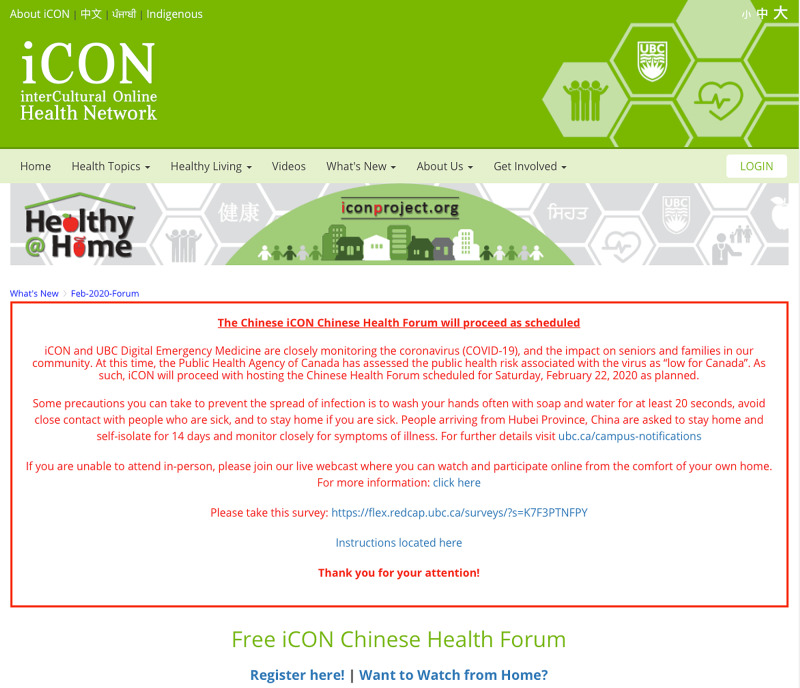
Official communication on COVID-19 developed in partnership with the British Columbia Ministry of Health for prospective forum attendees hosted on the interCultural Online Health Network's website Derived from the interCultural Online Health Network's website in February 2020. Available for public use. COVID-19: coronavirus disease 2019

We established a three-pronged approach for risk mitigation and prevention of spread prior to and at the forum. First, in the pre-forum phase, approximately two weeks leading up to the event, we made a traditional Chinese translated official communication on COVID-19, developed in partnership with the BC MoH, available on our program website (Figures 1-2). This communication brief was strategically placed on our website, such that prospective participants would encounter this information prior to completing their registration for the forum. The message was designed to raise awareness about reducing the spread of COVID-19, highlighting information on the current risk to the public from COVID-19, precautions to employ at home, and reminded people to self-monitor if they had recently traveled to China. Simultaneously, we assessed the need to promote our live webcast option more, which has been traditionally offered as an alternative to older adults who cannot attend the forum in-person due to mobility issues. The live virtual conference webcast was set up in three languages, including English, Cantonese, and Mandarin, with live interpretation. Our purpose in providing the BC MoH communication brief and leveraging the use of our language-specific live virtual conference option more was to help potential attendees understand the seriousness of the public health crisis and opt for remote attendance while providing older Chinese adults with the opportunity to benefit from the forum. Through these measures, we particularly emphasized the need for people who had recently traveled to the Hubei province of China (based on the official messaging of that time) to avoid attending in-person and reduce the risk of disease spread at the forum.

**Figure 3:**
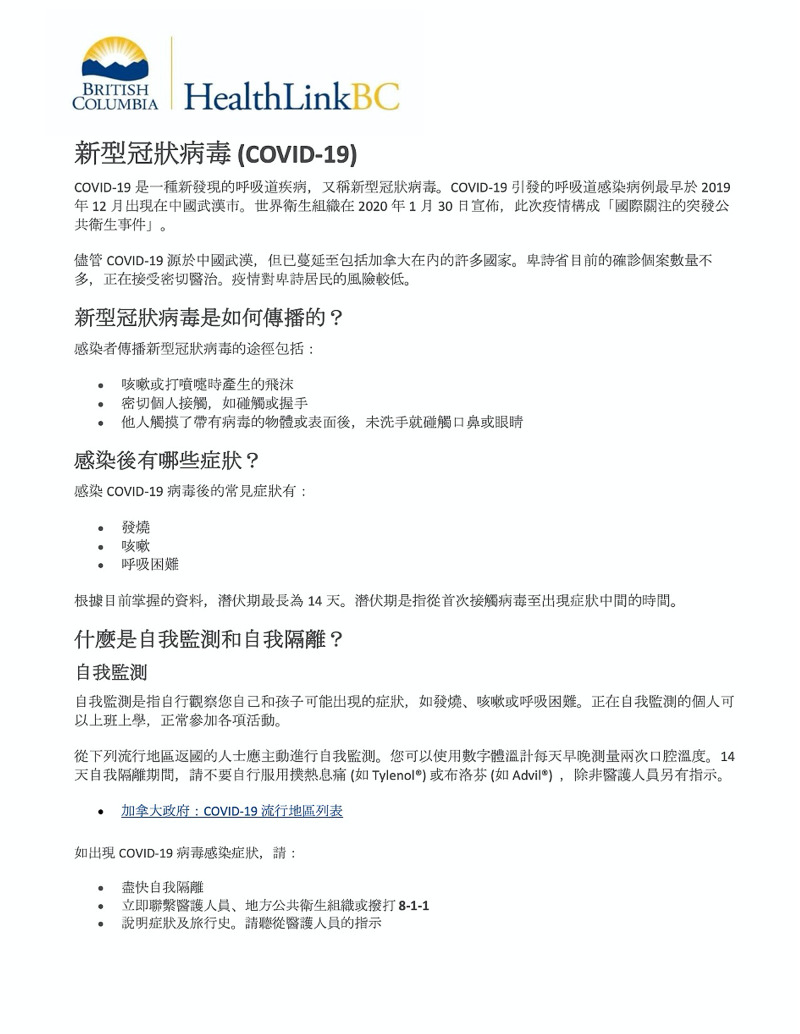
Excerpt from the traditional Chinese-translated version of the health literature developed by the British Columbia Ministry of Health highlighting information on COVID-19 provided as part of the participant welcome package at the interCultural Online Health Network's 2020 Chinese Forum Derived from the British Columbia Ministry of Health's website in February 2020. Available for public use. COVID-19: coronavirus disease 2019

**Figure 4:**
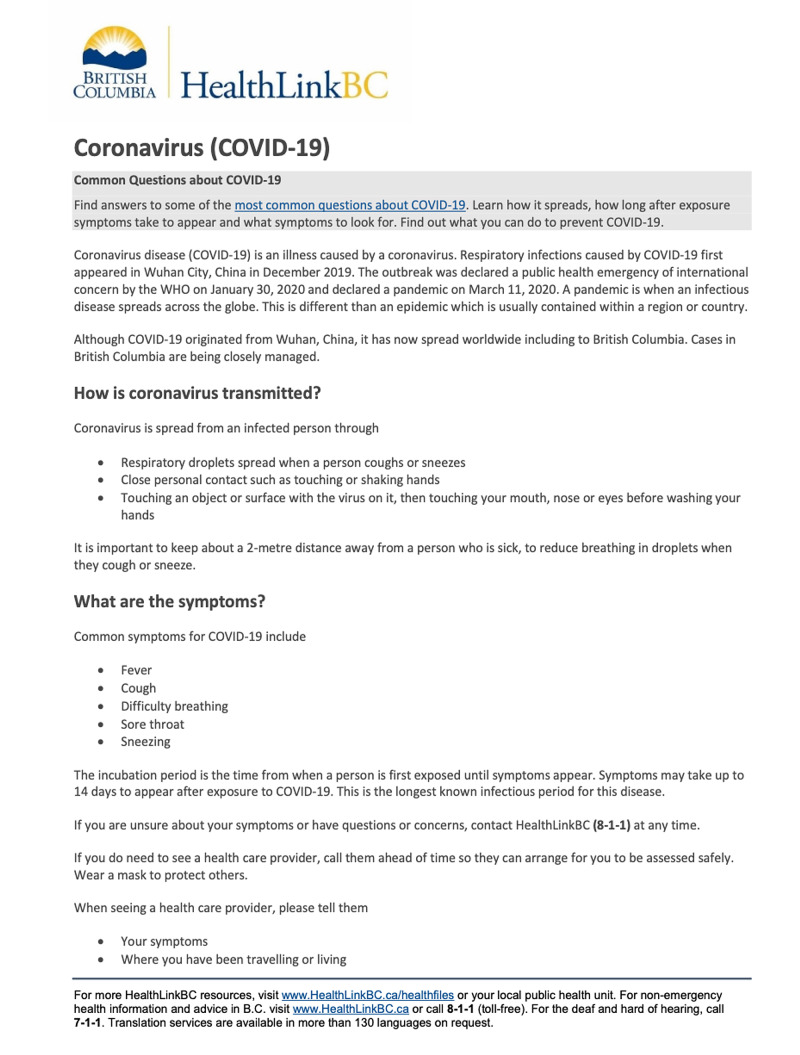
Excerpt from the health literature developed by the British Columbia Ministry of Health highlighting information on COVID-19 provided as part of the participant welcome package at the interCultural Online Health Network's 2020 Chinese Forum Derived from the British Columbia Ministry of Health's website in February 2020. Available for public use. COVID-19: coronavirus disease 2019

At the forum, we provided traditional Chinese translated health literature produced by the BC MoH to all our participants, as well as an English version, as part of their registration package (Figures 3-4). This document included extensive information on the virus itself, how to prevent spread, and guidelines on self-isolation based on travel history. Our purpose behind providing this document was to ensure participants were well-informed on the epidemic through official messaging. In addition, we had a dedicated time set for attendees to ask our health professional speakers their COVID-19-related questions during the question-and-answer session. We acknowledged the anxiety and uncertainty that our participants faced regarding an emerging public health crisis that was still developing, yet prevalent, among their community at the time. Thus, we had notified our health professional speakers to anticipate such questions prior to the forum and also had an official public health expert from VCHA specifically address the audience on COVID-19. 

Finally, we ensured that every participant sanitized their hands at the registration desk prior to entering the forum hall and had access to an adequate number of hand sanitizers throughout the forum hall. In addition, we ensured that every attendee received their own separate snack package at the registration desk to minimize person-to-person contact during the forum. 

## Results

There were 381 older Chinese adults who attended the 2020 iCON Chinese Health Forum in Vancouver, BC on February 22, 2020 (Table 1). From the total number of participants, 231 attended the forum in-person at the Chinese cultural centre, while 150 attended remotely through the live virtual conference option. This was a two-fold increase in online attendance compared to previous years, on average (74 in 2018 and 73 in 2016). 

**Table 1:** Number of Participants Attending iCON Chinese Health Forums Conducted Under the Theme of "Healthy @ Home" Over the Years

Attendees	2020		2018		2016	
	Number	%	Number	%	Number	%
In-person	231	60.6	279	79.0	269	78.7
Online	150	39.4	74	20.1	73	21.3
Total	381	100	353	100	342	100

Of the 93 participants that completed the post-forum survey, 89% self-reported that attending the forum would help them improve their overall health and quality of life to some degree. Finally, 87% of the participants reported that they learned about community resources and services that they can access to manage their chronic conditions as a result of attending the forum. 

To our knowledge, none of the participants had visible signs or symptoms of COVID-19. Although we did not follow-up with forum attendees regarding transmission after the event, we have not been contacted by the VCHA or the BCCDC regarding concerns of community spread at our forum. Thus, none of the participants are suspected to have contracted COVID-19 as a result of attending the forum. 

## Discussion

Using the February 22, 2020 iCON Chinese Health Forum as an example, we call attention to practical and local official guidelines-based risk mitigation tools that can be implemented to efficiently conduct a mass gathering during a crisis, without sacrificing the public benefit from the event. Our 2020 iCON Chinese Health Forum attracted a similar amount of people compared to previous years (ever since the onset of the “Healthy @ Home” theme) with twice as many participants opting to participate via the online option than previously. 

The modest attendance (overall and in-person) of older Chinese adults at the forum may be attributable to our effective risk mitigation strategies. First, our use of a culturally specific communication strategy prior to and during the forum may have served to reinforce their confidence in our organization. In fact, according to the WHO, proactive communication in times of crisis is one of the most important ways of mitigating conflict and building trust [20]. The official communication from BC MoH we made available on our website prior to the forum and the health literature from the BC MoH given out at the forum were both translated into traditional Chinese. Seeing their cultural beliefs represented, our participants may have been more likely to feel safe while attending and act on that information [21]. In fact, given the now well-documented disproportionate amount of burden shared by ethnic minorities with regards to COVID-19, it becomes even more important to emphasize the need for culturally tailored programming when passing on health messages or to promote safe practices when conducting mass gatherings in the future [22-23].

Second, the increase in the number of people attending the 2020 iCON Chinese Health Forum online may suggest that our promotion of the language-supported live virtual conference option encouraged more people to attend and potentially avoid any non-essential contact with others. Most importantly, it may have provided a reasonable alternative for those who had traveled to China at the time to stay at home without sacrificing the benefit of receiving culturally-specific health education. In fact, since the outset of the pandemic, several groups have highlighted the advantages of virtual approaches for event organization. Perhaps the provision of an online virtual conference is something many mass gathering organizers can consider, given its cost-effectiveness, feasibility, and inclusivity, wherever appropriate [24-25]. Options to participate online could even be beneficial in maintaining public safety while organizing events as the world economy opens up during the next phase of the pandemic [26]. 

Third, our dedicated question and answer session with the healthcare experts at the forum may have allowed our participants to feel engaged - a critical step in alleviating confusion and misunderstanding during a crisis. Several recent studies, including WHO’s official guidelines on risk communication and community readiness to COVID-19, have emphasized the importance of appropriate strategies to communicate risk when organizing an event during a crisis [20, 26-27]. The WHO document highlights the importance of a transparent dialogue between healthcare experts and members of the public to clearly communicate the perception of risk involved, which may often differ between the two parties [20]. Yezli and Khan stressed the necessity of the involvement of official authorities in such communication to ensure compliance and uptake by the public [26]. This supports our approach for the 2020 iCON Chinese Health Forum. We ensured the public attending the forum were well-informed on the potential risks involved by organizing the question-and-answer session between experts and participants, in addition to providing official health literature from the VCHA at the forum. 

Finally, our provision of sanitizers and individual food packets throughout the forum was necessary, given that COVID-19 primarily spreads through droplets and contaminated hands/surfaces [28]. This was also in line with the WHO’s messaging around frequent hand washing and sanitizing as being the best ways to prevent infection and COVID-19 spread [29]. In fact, according to a recent paper by Pradhan et al. evaluating the best preventive care approaches to curbing infection spread, including COVID-19, hand sanitization has been cited as the second most important strategy, with disinfecting surfaces being the first [30]. Accordingly, making sanitizers widely accessible may be a good strategy to not only ensure participant safety but also environmental safety when organizing events during and after COVID-19 [25].

There are several broader implications of our approach, even though we are unable to empirically assess any direct effects of the precautionary measures and the risk mitigation strategies that we employed on the health status of the older Chinese adults who attended the forum. With only six cases in BC at that time, there were no official guidelines from the local health authorities that instructed organizers to screen or test participants for COVID-19 prior to or follow-up with them after mass gatherings. It is possible that the fact that there were no positive cases of COVID-19 which could be traced back to our forum is because no one who had tested positive attended the forum in the first place, reducing the possibility of potential spread. We cannot completely discount the possibility of chance in curbing the spread of infection at the forum. However, even if this is true, it further supports the effectiveness of the precautionary steps we had in place. Our practices of using official translated communications from the BC MoH, asking people who were symptomatic or had traveled to China recently to stay at home and participate online was unconventional at the time. Moreover, the forum’s effectiveness in improving older Chinese adults’ perceived ability to self-manage their chronic diseases is an important benefit that cannot be overlooked. A majority of attendees reporting a foreseeable benefit in their overall health and well-being, in addition to their understanding of accessible community resources, highlights the importance of this event and supports our decision to proceed with it by addressing foreseeable risks through the measures we took. 

Future events may benefit from adopting our risk mitigation strategies, which involved the dissemination of timely and culturally specific communication with our target audience, as safe practices when adapting to a local context during crises. Strategies, such as official communication from government and health authorities to inform attendees, options to participate online, and sanitizer provisions, could be beneficial ubiquitously, whenever appropriate. Based on the lessons learned from the forum, we also recognize that additional safety precautions may be warranted should an event be organized at another point in time. In fact, based on the current landscape and public health authorities’ guidance, we have already transitioned to a completely virtual programming strategy for subsequent iCON workshops and forums to maintain public safety while disseminating culturally specific health education. Widespread adoption of all such public health measures and adapting to the local threat to public safety will be essential for reinstating the public trust in authorities as the economy opens up and we move towards the next phase of this pandemic [27]. 

## Conclusions

This paper provides insights into practical and evidenced-based strategies that we employed to conduct a mass gathering at the outset of COVID-19 while upholding public interest in our event. Organizing the February 22, 2020, iCON Chinese Health Forum required careful consideration of local public health guidelines, adopting clear communication strategies that addressed attendee specific needs, and risk mitigation alternatives. 
